# A 3-Year-Old With Leg Pain After Visiting a Trampoline Park

**DOI:** 10.1016/j.acepjo.2025.100053

**Published:** 2025-01-31

**Authors:** Corey Horn, Lindsay Tjiattas-Saleski

**Affiliations:** 1 Edward Via College of Osteopathic Medicine Carolinas, Spartanburg, South Carolina, USA; 2 Prisma Health, Greenville, South Carolina, USA

**Keywords:** pediatrics, orthopedics, greenstick, valgus, tibia, fracture

## 1 Patient Presentation

**Figure 1:**
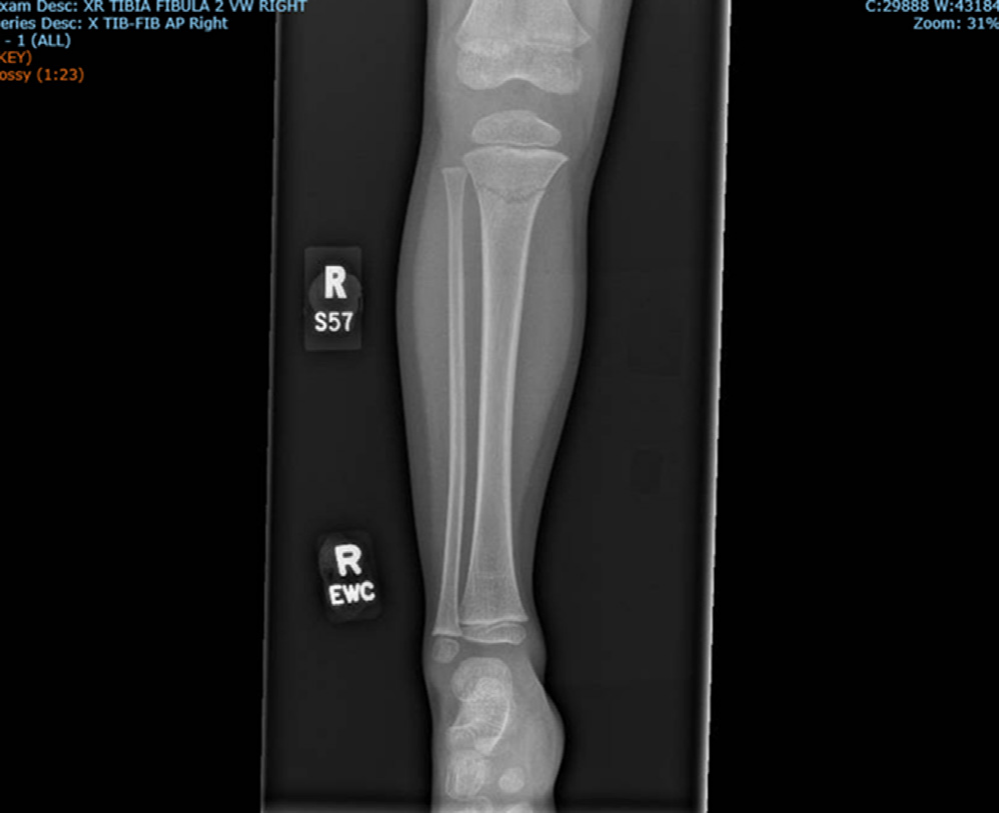
Anteroposterior plain film of right lower limb.

**Figure 2:**
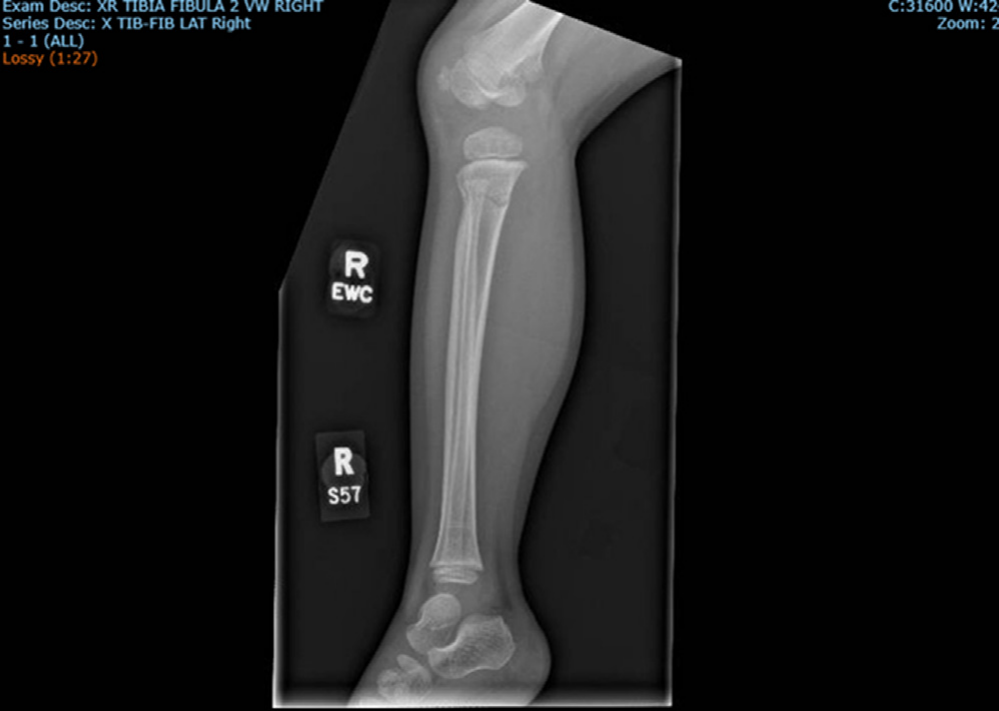
Lateral plain film of right lower limb.

A 3-year-old boy presents to the emergency department with a complaint of right leg pain. The onset of pain occurred roughly 24 hours prior when the patient accidentally collided with a family member while jumping on a trampoline. The exact positioning of the impact is unclear. The patient noted general lower leg pain without the ability to identify an exact location. The leg was tender, and the patient’s body weight was not tolerated while standing or ambulating. Over-the-counter pain medications were taken at home but provided no relief. No other injury or illness was revealed by the patient or guardian. Plain film x-ray imaging of the right lower leg was ordered and shown below in Figures 1 and 2.

## 2 Diagnosis: Cozen’s Fracture

Plain film imaging revealed a nondisplaced right proximal metaphyseal tibial fracture, less commonly referred to as a “Cozen’s Fracture.”^1^ Proximal tibial fractures are seen most in children 3 to 6 years of age and are associated with a condition known as “Cozen’s phenomenon.”^2^ Cozen’s phenomenon is a valgus tibial deformity as a result of improper healing in fractures of the proximal tibia including greenstick and complete fractures.^2^^,^^3^ Proper angulation in the reduction of tibial fractures is thought to reduce the risk of valgus deformity, although it is unclear how influential initial management is in long-term avoidance as other variables such as body weight and fracture displacement contribute to the incidence of Cozen’s phenomenon.^2^, ^3^, ^4^ Long leg casts in extension with varus mold is the management of choice to prevent the valgus deformity, especially when the fibula remains intact.^2^^,^^4^ Cozen’s fractures are commonly cited as injuries related to trampoline use with proximal tibia fractures accounting for 23% of trampoline-related fractures.^4^ Proximal tibia fractures in 3- to 6-year-old patients should be monitored closely, as there is a tendency for progression to Cozen’s valgus deformity, but the phenomenon typically resolves through observation alone.^4^^,^^5^

## Conflict of Interest

All authors have affirmed they have no conflicts of interest to declare.
